# Obesity, diabetes, and cancer: epidemiology, pathophysiology, and potential interventions

**DOI:** 10.20945/2359-3997000000647

**Published:** 2023-06-19

**Authors:** Leonardo de Andrade Mesquita, Laura Fink Wayerbacher, Gilberto Schwartsmann, Fernando Gerchman

**Affiliations:** 1 Universidade Federal do Rio Grande do Sul Programa de Pós-graduação em Ciências Médicas: Endocrinologia Porto Alegre RS Brasil Programa de Pós-graduação em Ciências Médicas: Endocrinologia, Universidade Federal do Rio Grande do Sul, Porto Alegre, RS, Brasil; 2 Hospital de Clínicas de Porto Alegre Porto Alegre RS Brasil Hospital de Clínicas de Porto Alegre, Porto Alegre, Brasil, Porto Alegre, RS, Brasil; 3 Universidade Federal do Rio Grande do Sul Faculdade de Medicina Porto Alegre RS Brasil Faculdade de Medicina, Universidade Federal do Rio Grande do Sul, Porto Alegre, RS, Brasil

**Keywords:** Cancer, obesity, diabetes, weight loss

## Abstract

The proportion of deaths attributable to cancer is rising, and malignant neoplasms have become the leading cause of death in high-income countries. Obesity and diabetes are now recognized as risk factors for several types of malignancies, especially endometrial, colorectal, and postmenopausal breast cancers. Mechanisms implicated include disturbances in lipid-derived hormone secretion, sex steroids biosynthesis, hyperinsulinemia, and chronic inflammation. Intentional weight loss is associated with a mitigation of risk for obesity-related cancers, a phenomenon observed specially with bariatric surgery. The impact of pharmacological interventions for obesity and diabetes is not uniform: while metformin seems to protect against cancer, other agents such as lorcaserin may increase the risk of malignancies. However, these interpretations must be carefully considered, since most data stem from bias-prone observational studies, and high-quality randomized controlled trials with appropriate sample size and duration are needed to achieve definite conclusions. In this review, we outline epidemiological and pathophysiological aspects of the relationship between obesity, diabetes, and malignancies. We also highlight pieces of evidence regarding treatment effects on cancer incidence in these populations.

## INTRODUCTION

Obesity, especially central and visceral obesity, and associated metabolic disorders such as diabetes mellitus, are established risk factors for cardiovascular disease – including ischemic heart disease and cerebrovascular disease –, the leading cause of death worldwide and the most common cause of disability-adjusted life years (DALYs) (1–3).

The relationship between obesity, diabetes, and their precursors (overweight, insulin resistance, prediabetes) and the development of cancer has been increasingly recognized (4). Such association grows in relevance when we factor into it the rising proportion of deaths due to cancer, with malignant neoplasms leading the causes of death in high-income countries (5). Moreover, the proportion of deaths due to cancer has increased in subjects with diabetes over time, while cardiovascular-related mortality is declining (6–8).

In this narrative review, we analyze the links between overweight, obesity, insulin resistance, prediabetes, diabetes, and cancer from the epidemiological and pathophysiological aspects and the impact of nonpharmacological, pharmacological, and surgical treatments for these diseases in cancer development and mortality.

## EPIDEMIOLOGICAL ASPECTS

The prevalence of overweight (defined as body mass index (BMI) ≥ 25 and < 30 kg/m² in adults) and obesity (BMI ≥ 30 kg/m²) has risen to worrying levels of 35%-40% and 10%-15% worldwide, respectively, with mean population BMI increasing around 0.6 kg/m² every decade between 1975 and 2014. The prevalence is higher in women and high-income countries compared with low- and middle-income ones. If the trend continues, the global prevalence of obesity could reach about 20% from 2025 to 2030 (9–11).

Diabetes, which has overweight and obesity as major risk factors, closely follows the trend: its global prevalence in adults (aged 20-79) was estimated at 10.5%, representing 536.6 million people, with projections estimating this number could reach 783.2 million by 2045, comprising 12.2% of the estimated population, as obesity prevalence and mean population age increase. The current age-standardized prevalence of diabetes is higher in middle-income countries, and the greatest increase in the following decades is expected to come from this group. As a costly disease to manage, with current global diabetes-related health expenditures estimated at 966 billion US dollars, the rise in prevalence imposes an even higher load on national health systems (12).

### Epidemiological association between obesity and cancer

Accumulated evidence indicates that obesity in adults is associated with an elevated risk of at least 12 malignant neoplasms, namely: endometrial, post-menopausal breast, ovarian, esophageal (adenocarcinoma), gastric (cardia), colorectal, pancreatic, liver, gallbladder, kidney, thyroid, and multiple myeloma. The strongest relationships are described for endometrial and esophageal adenocarcinoma, whose relative risk in individuals with obesity compared with those with normal BMI exceeds 4 (13–15). Using 2012 cancer incidence data from GLOBOCAN, almost 4% of the reported cases of cancer could be attributed to high BMI (16).

While BMI remains the fundamental criteria to define overweight and obesity, it does not adequately represent adiposity (particularly visceral adipose tissue, which is more metabolically active and has greater implications in the relationship between obesity and cancer) (13,17). Other measures such as waist circumference, waist-to-height ratio, and waist-to-hip ratio, as well as advanced characterizations of central fat deposition such as visceral adipose tissue (quantified by computed tomography) and trunk-to-peripheral fat ratio (evaluated by dual-energy X-ray absorptiometry scan), also show a direct relationship with the incidence of malignant neoplasms (18–22). A recent study from the UK Biobank found similar associations between different adiposity markers and cancer incidence (23).

Overweight or obesity in adulthood lead to an increased risk of cancer. Notably, excess weight in childhood and adolescence has also been associated to a greater incidence of certain malignant neoplasms (24–26). Moreover, large cohort studies have demonstrated that weight gain in early or mid-adulthood have a direct relationship with obesity-related cancer in later ages (27–30). Even so-called ‘metabolic healthy obesity’ (high BMI in the absence of metabolic abnormalities related to obesity) seems to lead to a higher risk of malignant neoplasms (31).

Cancer mortality follows cancer incidence and increases with higher BMI in the general population (25,32). In the overall cohort of cancer patients, a high BMI at diagnosis is associated with worse outcomes. Notwithstanding, for a subset of neoplasms such as renal cell carcinoma, lung cancer, melanoma, and metastatic cancer in older patients, obesity has an inverse relationship with mortality (33,34). While biological mechanisms, including lesser aggressiveness of some neoplasms in obesity and greater nutritional reserve to undergo antineoplastic therapy could be implied, the most likely explanation for this “obesity survival paradox” in cancer involves methodological issues such as the limitations of using BMI as diagnostic criteria for obesity, residual confounding by factors such as tobacco smoking and reverse causality (weight loss due to cancer cachexia, being more pronounced in advanced tumors) (13,35,36).

Notably, obesity is inversely correlated with the risk of premenopausal breast cancer (15,17).

### Epidemiological association between diabetes and cancer

Based on evidence from observational and Mendelian randomization studies type 2 diabetes has been implied as a risk factor for several malignancies, including postmenopausal breast, endometrial, colorectal, pancreatic, gallbladder, and liver cancers alongside obesity (37–39). In a large cohort, more than 2% of cancer cases could be attributed to diabetes alone (16). Not only individuals with diagnosed diabetes show this association, but also people with increased plasma glucose in the prediabetic range, a stage in which metabolic phenomena, such as insulin resistance, are already present (40,41). Likewise, a possible association between type 1 diabetes and an increased risk of cancer has been suggested, but the evidence is limited (17,42).

Most diagnoses of cancer in subjects with type 2 diabetes occur in the three months following the diagnosis of diabetes, decreasing thereafter (43). While this finding may be partially related to detection bias or reverse causality (development of hyperglycemia due to the underlying malignancy), the diagnosis of type 2 diabetes is usually preceded by a long period of insulin resistance and hyperinsulinemia, which are potential mechanisms underlying the association with cancer (17,41).

In large cohort studies and a recent meta-analysis, people with type 2 diabetes had a 20%-30% higher chance of dying from cancer compared with subjects without diabetes (39,44–46). Among patients with malignancies, preexisting diabetes confers an increased risk of all-cause mortality, but the impact may be greater on noncancer (mainly cardiovascular) deaths than on cancer-specific mortality (17,47).

Prostate cancer is a notable exception in the relationship between diabetes and malignancies, similar to premenopausal breast cancer and obesity. In several studies and systematic reviews in Western populations, diabetes is associated with a lower incidence of prostate cancer (38,39,44,48–52). The causality of this association is still debated, considering a possible detection bias resulting from low circulating prostate-specific antigen in men with diabetes (17,39,49).

### Pathophysiological aspects

**Figure 1:**
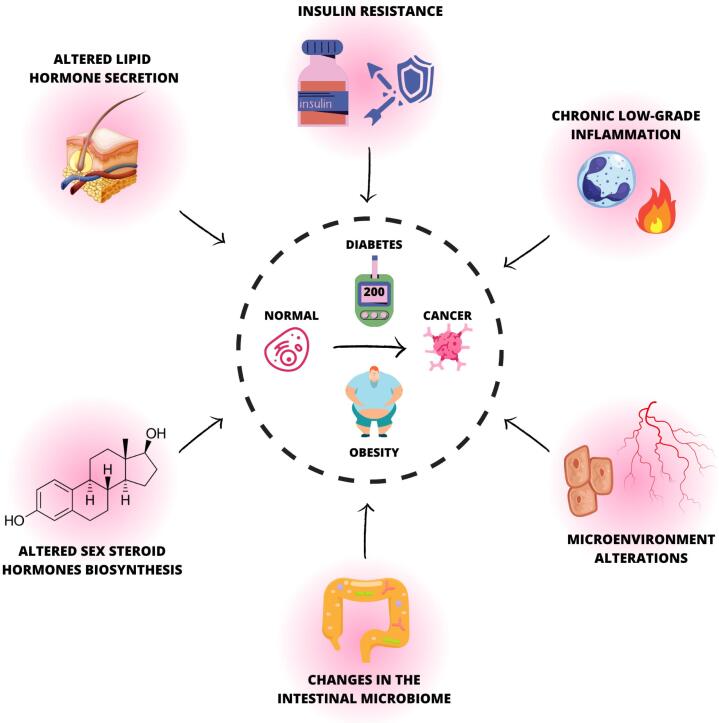
Mechanisms linking obesity and diabetes with cancer. The Figure 1 shows mechanisms linking obesity, diabetes, and cancer: metabolic derangements (such as altered sex steroid hormones biosynthesis, altered lipid hormones secretion, insulin resistance, and subsequent hyperinsulinemia, hyperglycemia), chronic low-grade subclinical inflammation, alterations in the tumor microenvironment, and changes in the gut microbiome.

The mechanisms linking obesity, diabetes, and cancer include metabolic derangements (such as alterations in lipid-based hormone secretion, biosynthesis of sex steroid hormones, insulin resistance, and subsequent hyperinsulinemia, hyperglycemia), and chronic low-grade subclinical inflammation (13,17,41,53–56) (Figure 1).

Obesity not only increases white adipose tissue, but also alters the adipocyte endocrine function: reducing adiponectin synthesis and increasing leptin production. These peptide hormones have antagonistic actions regarding carcinogenesis: leptin may act as a growth factor and promotes an inflammatory environment, while adiponectin suppresses cancer cell proliferation and decreases proinflammatory mediators (17,57,58). In a systematic review and meta-analysis of observational studies, adiponectin, and leptin had inverse and direct relationships, respectively, with the risk for specific subtypes of cancer (59).

Insulin resistance – a condition frequently observed in people with obesity, and fundamental in the pathophysiology of type 2 diabetes – promotes compensatory hyperinsulinemia, at least when the β-cell function is preserved (17). Insulin is an anabolic hormone that acts through tyrosine-kinase membrane receptors, leading to mitogenic and anti-apoptotic effects, particularly in cancer cells that lose downregulation of the insulin receptor (60). Hyperinsulinemia also stimulates insulin-like growth factor-1 (IGF) production in the liver and reduces circulating levels of IGF-binding proteins, culminating in increased plasma levels of IGF-1 and IGF-2, two growth factors that promote cell proliferation (17,54,57,58). When pancreatic β-cells are unable to produce more insulin in face of increasing insulin resistance, hyperglycemia ensues. High levels of plasma glucose could theoretically favor cancer development since neoplastic cells shift their energy metabolic pathways to anaerobic glycolysis, a process that requires greater amounts of glucose moieties to produce the same energy as oxidative phosphorylation (60,61). However, since hyperglycemia indirectly promotes hyperinsulinemia and elevated IGF levels, its causal role in carcinogenesis is still debated, and the weaker association of type 1 diabetes with the risk of cancer supports the idea that hyperglycemia may not be a key-contributing mechanism (13,17).

Adipocytes peripherally convert androgens to estrogens by the action of aromatase. The greater white adipose tissue volume and the increased expression of aromatase within individual adipocytes induced by proinflammatory cytokines, combined with decreased levels of sex hormone-binding globulin, lead to higher circulating levels of estrogens, steroid hormones implicated in the development of breast and endometrial cancers (13,17,54,58). Ovarian hyperandrogenism leading to chronic anovulation, and consequently decreased progesterone levels, also contributes to the development of endometrial cancer (due to the unopposed estrogen effect) and may explain the decreased incidence of premenopausal breast cancer (13,62).

In obesity, white adipose tissue shows increased infiltration by immune cells such as macrophages, that secrete proinflammatory cytokines such as tumor necrosis factor alpha and interleukins 1β and 6, whose production is also stimulated by the described changes in adiponectin and leptin. These inflammatory mediators not only contribute to insulin resistance and increased peripheral estrogen conversion but also activate signaling pathways implicated in tumor development such as the JAK/STAT pathway (13,17,54,57).

Other potential mechanisms include alterations in the tumor microenvironment, increased oxidative stress, changes in the gut microbiome, and mechanical factors (such as increased gastroesophageal reflux leading to Barret's esophagus and consequently to esophageal adenocarcinoma) (13). However, studies regarding the exact mechanisms linking obesity, diabetes, and cancer are still necessary.

### Impact of obesity and diabetes treatments

#### Intentional weight loss and glycemic control

Consistent with evidence attributing a higher risk of cancer with increasing BMI and weight gain during early and middle adulthood, investigations regarding weight loss suggest a possible reduction in the incidence of obesity-related cancer (particularly female-specific cancers), but the evidence is primarily based on observational studies (13,17,63).

The Iowa Women's Health Study in 2003 and the Women's Health Initiative Observational Study in 2019, both cohort studies with women, reported reductions of 14% and 12% in obesity-related cancer risk (especially breast, colon, and endometrial) with reductions of at least 9 kg and 5% of initial weight, respectively (64,65). A systematic review in 2012 retrieving almost exclusively observational studies found that 16 of 34 studies reported an inverse relationship between intentional weight loss and cancer incidence, and no study reported an opposite effect (66). Regarding randomized trials, a meta-analysis from 2017 found only very low-quality evidence from dietary trials targeting weight loss, showing a trend for lower cancer mortality and no effect on the risk of new cancers (67). More recently, follow-up data from the Look AHEAD randomized controlled trial found a trend toward a lower incidence of obesity-related cancers in subjects assigned to an intensive lifestyle intervention for weight loss, but without achieving statistical significance (possibly due to lack of statistical power) (68).

Unlike weight loss, tight glycemic control, at least in the short and medium term, seems to have only a weak or even null association with cancer incidence (69,70).

#### Metformin

Metformin is the recommended first-line medicine for most patients with type 2 diabetes because it is safe, inexpensive, and effective in reducing glycated hemoglobin, while potentially protecting against cardiovascular disease and mortality and promoting modest weight loss (71).

Due to its mechanisms of action, including AMPK activation and mTOR inhibition, and its metabolic consequences, such as decreased insulin resistance and weight loss, it has been hypothesized that metformin may have a role in cancer prevention (72). Since the publications of a case-control study in 2005 and a cohort study from the same authors in 2009 suggesting an inverse association between the use of metformin and cancer in people with type 2 diabetes (73,74), data from subsequent observational research has supported that finding (75,76), whereas secondary analyses from randomized controlled trials do not confirm such relationship (77,78). This divergence may result from biases inherent to observational studies (79), but the randomized trials included in the meta-analyses were not specifically designed to assess cancer as an outcome, they were conducted on populations that were not at increased risk for malignancies, and had short follow-up periods (72). Awaited reports on cancer incidence from extended periods of randomized trials such as the Diabetes Prevention Program Outcomes Study (80) could still influence these conclusions.

#### Thiazolidinediones

Thiazolidinediones are agonists of the peroxisome proliferator-activated receptor γ used as an additional therapy for type 2 diabetes due to their effect on reducing insulin resistance (81). Their use in clinical practice is challenged by adverse effects such as weight gain, edema, bone fractures, and heart failure (82). While a meta-analysis found a small reduction in cancer incidence with thiazolidinediones (78) since the observation of more bladder cancer cases in the pioglitazone group in the PROactive trial in 2005 (83), several meta-analyses of observational studies have described a small, but significant increase in bladder cancer risk with thiazolidinediones, and the drug has been withdrawn from the market in countries such as Germany, France, and India (84,85).

#### Sodium-glucose cotransporter-2 (SGLT-2) inhibitors

SGLT-2 inhibitors have been recently introduced in diabetes management and are recommended as either first-line therapy or in combination with metformin for patients with type 2 diabetes and high cardiovascular risk, heart failure, or chronic kidney disease with albuminuria (71). A 2017 meta-analysis including 46 randomized trials found no significant association between the use of SGLT-2 inhibitors and the overall risk of cancer, but described an increased risk of bladder cancer, particularly with empagliflozin (86). However, this finding was mostly based on data from the EMPA-REG OUTCOME trial, and after analyzing only cases with onset after six months of therapy, the number of events was small to undertake such a conclusion (87,88). In fact, a subsequent meta-analysis including only trials longer than one year found no difference in the incidence of malignancies between SGLT-2 inhibitors and comparator interventions (89).

#### Incretin-based drugs

Incretin-based drugs comprise dipeptidyl peptidase-4 inhibitors (DPP-4is) and glucagon-like peptide-1 receptor agonists (GLP-1RAs). Current guidelines recommend GLP-1RAs as initial therapy or in combination with metformin for patients with type 2 diabetes and high cardiovascular risk (71), and two GLP-1RAs, liraglutide and semaglutide, are approved for the treatment of obesity after results from large clinical trials (90,91). DPP-4is may be used as additional therapy after metformin and/or SGLT-2 inhibitors (but not GLP-1RAs), are weight neutral, and pose minimal hypoglycemia risk (71).

Firstly, early observational studies based on reported cases demonstrated potential side-effects such as increases in medullary thyroid and pancreatic malignancies due to biological plausibility and elevations in cancer-associated biomarkers (92–95). The publication of the SCALE trial was associated to another concern, breast cancer, as more cases were identified in the liraglutide group (90). Several meta-analyses have examined the relationship between incretin-based therapies and both site-specific and overall cancer risk and reported no association between the drugs and the risk for malignant neoplasms (96–101). Although an observational study using data from the UK Clinical Practice Research Datalink and pharmacovigilance data from VigiBase suggested an association between DPP-4is and possibly GLP1-RAs with cholangiocarcinoma, few cases have been reported in clinical trials, not allowing for a definite conclusion (100,102).

#### Insulin and insulin secretagogues

The role of hyperinsulinemia in tumorigenesis arises the question of whether the administration of exogenous insulin or stimulation of endogenous insulin production induces cancer development in humans, which showed controversial results (103).

Observational studies published in the 2000s suggested that insulin users had a higher risk of malignant neoplasms, particularly colon, breast, and pancreatic cancers (104–108). The reported risk was higher with long-acting insulin analogs, especially insulin glargine. Both these studies and subsequent reports were criticized due to methodological flaws or biases (109–111). Meta-analyses examining this topic have found conflicting results, but even those reporting a possible increased risk of cancer with exogenous insulin, acknowledge that the quality of the included studies does not allow for a definite conclusion (78,112–115).

Regarding insulin secretagogues (sulfonylureas and meglitinides), similar findings have been reported; meta-analyses of observational studies suggest an increased incidence of cancer, but evidence from randomized trials indicates no association (78,116).

#### Lorcaserin

Lorcaserin is a selective agonist of the 5-HT2C serotonin receptor approved in 2012 by the FDA for the treatment of obesity based on three randomized controlled trials (117–119). While both these studies and the large CAMELLIA-TIMI 61 trial reported no imbalance in neoplasms with lorcaserin compared with placebo, a FDA review with follow-up data from this trial found a disproportional (although non-significant) increase in cancer cases in the lorcaserin group, motivating the market withdrawal of the drug (120,121). Combining the results of the four studies, a meta-analysis from our group found similar results but these results were heavily influenced by the CAMELLIA-TIMI 61 study, which provided most of the cases due to its larger sample size and follow-up period (122).

The example of lorcaserin reinforces the need for longer follow-up for randomized trials to adequately assess cancer outcomes, as the difference between groups only started after six months of treatment and increased with a longer exposure (121).

#### Bariatric surgery

Bariatric surgery, usually performed as gastric bypass or sleeve gastrectomy, is the most effective treatment for obesity, leading to greater and more sustained weight loss and better control of comorbidities, including type 2 diabetes, when compared with lifestyle and medical treatments (123).

Data from observational studies, compiled in several meta-analyses, indicate a reduction from 30% to 50% in the incidence of malignancies in subjects that underwent bariatric surgery (124–129). Agreeing with evidence from the relationship between weight loss and the risk of cancer, the effect is most pronounced in women, particularly for sexual hormone-related neoplasms. As with pharmacological interventions, these inferences are based on observational data and should be considered carefully due to inherent biases from this study design.

In conclusion, the role of obesity and diabetes as risk factors for cancer development has become progressively clearer. Weight loss seems to reduce the risk of obesity-associated cancers, particularly female-specific neoplasms. Further research, with high-quality studies (preferably randomized controlled trials with long follow-up), is needed to validate interventions that attenuate this risk, with metformin and bariatric surgery being the most promising candidates.
